# Relatively Low Rate of Heterotopic Ossification Following Primary Total Knee Arthroplasty: A Systematic Review and Meta-analysis

**DOI:** 10.5435/JAAOSGlobal-D-21-00096

**Published:** 2021-07-22

**Authors:** Ioannis Gkiatas, William Xiang, Theofilos Karasavvidis, Eric N. Windsor, Michael-Alexander Malahias, T. David Tarity, Peter K. Sculco

**Affiliations:** From the Stavros Niarchos Foundation Complex Joint Reconstruction Center, Hospital for Special Surgery, New York, NY (Dr. Gkiatas, Mr. Xiang, Mr. Windsor, Dr. Malahias, Dr. Tarity, and Dr. Sculco), and the School of Medicine, Faculty of Health Sciences, Aristotle University of Thessaloniki, Thessaloniki, Greece (Mr. Karasavvidis).

## Abstract

Heterotopic ossification (HO) is abnormal growth of ectopic bone and negatively affects the outcomes after total knee arthroplasty (TKA). This systematic review and meta-analysis were performed to characterize the prevalence and severity of HO after primary TKA.

**Methods:**

A systematic review of the literature was performed according to the Preferred Reporting Items for Systematic Reviews and Meta-Analyses guidelines. Patient demographics, publication year, and HO prevalence after a primary TKA were recorded. A meta-analysis was performed to determine the overall prevalence of HO formation, and a subanalysis compared the studies published in different timeframes to determine whether a temporal effect exists for HO prevalence.

**Results:**

Two thousand nine hundred eighty-eight patients underwent primary TKA across the included studies. Fourteen percent of patients (9% to 20%; I^2^: 93.68%) developed HO postoperatively during a mean follow-up of 40.1 months (11 to 108 months). HO rates seemed to decrease in studies published in more recent years, with a pooled HO prevalence of 5% (0% to 13%; I^2^: 92.26%) among studies published in the past 15 years compared with 18% (12% to 25%; I^2^: 92.49%) among studies published before then.

**Conclusion:**

Although studies reported a relatively low overall rate of HO after a primary TKA, the absence of a single, standardized classification system precludes the comparisons of HO severity between studies. Overall, HO prevalence seems to have decreased over time, likely reflecting the changes in perioperative medication protocols.

Heterotopic ossification (HO) is the abnormal formation of well-circumscribed extraskeletal bone in soft tissue and is commonly seen after trauma, such as spinal cord injury, traumatic brain injury, burns, and fractures.^1^ HO is also a widely reported sequela of surgery, specifically total joint arthroplasty (TJA), and previous reports have documented the presence of HO development after a variety of TJA procedures.^2,3,4,5^

Because of the potential negative clinical ramifications of HO, it is valuable for surgeons to understand the risk of HO development after an arthroplasty procedure. To this end, previous systematic reviews have collated studies of total elbow, ankle, cervical disk, and hip arthroplasties and reported the overall postoperative rates of HO after these procedures.^2,3,4,5^ However, although the total knee arthroplasty (TKA) is a commonly performed procedure, no summative assessment exists in the literature reporting on the postoperative development of HO and its implications after TKA. Thus, the objective of this systematic review and meta-analysis was to perform a comprehensive analysis on the reported prevalence of HO after a TKA.

## Methods

Data collection for this systematic review was conducted in accordance with the Preferred Reporting Items for Systematic Reviews and Meta-Analyses guidelines.^6^ The U.S. National Library of Medicine (PubMed/MEDLINE) and the Cochrane Database of Systematic Reviews were queried for publications from January 1971 to October 2020 by using the following keywords: “(Heterotopic) OR (Ectopic) AND (Total Knee).”

The inclusion criteria consisted of (1) studies describing human subjects of any age or sex, (2) studies including >10 patients, and (3) studies reporting the prevalence rates of HO after primary TKA surgeries. The exclusion criteria consisted of (1) review articles, (2) case studies, (3) non-English language publications, (4) studies reporting on the prevalence rates of HO after revision TKA surgeries only, and (5) studies where prophylactic radiation was administered to prevent the development of HO.

The methodological quality of each study and the different types of detected bias were assessed by a single reviewer (W.X.) via the modified Coleman methodology score. One reviewer (I.G.) subsequently reviewed all studies to perform standardized data collection, followed by independent assessment by another reviewer (T.K.) to assess for accuracy. Data collected from each article included demographic information, consisting of sex, mean age, mean follow-up time, year of publication, and reported the prevalence rates of HO after primary TKA. Both reviewers compared the data collected, and all disagreements were resolved by discussion.

Continuous variables were estimated as mean ± SD, whereas categorical variables were reported as relative frequencies. A random-effects meta-analysis of proportions was undertaken to estimate the overall prevalence of HO in patients who underwent a primary TKA. The I-squared statistic was used to assess heterogeneity, with I^2^ values greater than 50% indicating notable heterogeneity.^7^ Forest plots were used to graphically display the effect size in each study and the pooled estimates, with *P* values <0.05 considered statistically significant. STATA 14.1 (StataCorp) was used as the statistical software.

## Results

**Figure 1:**
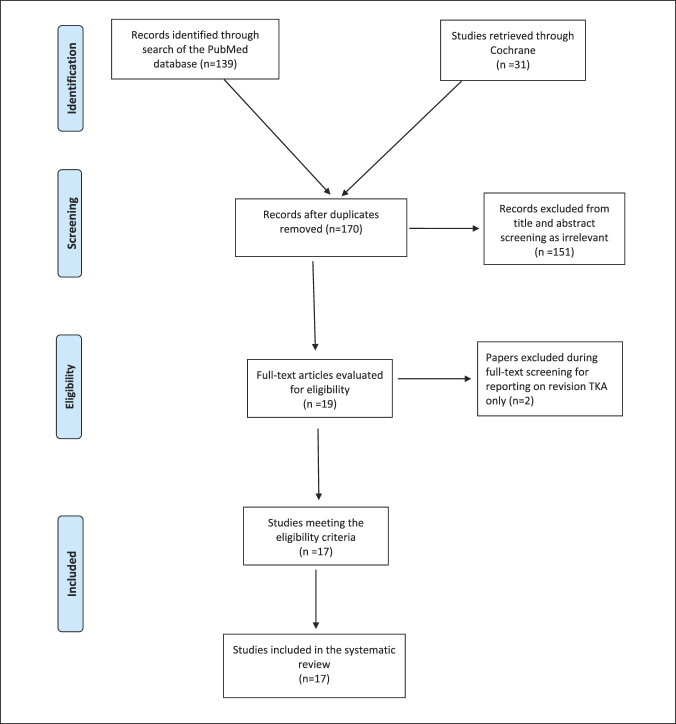
Flow chart diagram.

The initial literature search yielded 170 potentially relevant records after duplicates were removed. After screening titles and abstracts, 19 articles were retrieved for full-text evaluation. Two articles were excluded at this stage for reporting on revision TKA procedures only, leaving 17 studies that met the predetermined eligibility criteria and were included in the final meta-analysis^8,9,10,11,12,13,14,15,16,17,18,19,20,21,22,23,24^ (Figure 1). Fourteen of these studies were retrospective cohort studies, whereas the other three^8,11,12^ were prospective cohort studies. The mean modified Coleman score of this review was 54.5 ± 7.5, which indicates moderate overall study methodology.

In total, 2988 primary TKA patients were included in this meta-analysis.^8,9,10,11,12,13,14,15,16,17,18,19,20,21,22,23,24^ The mean duration of follow-up was 40.1 ± 27.8 months, ranging from 11 to 108 months. The mean patient age was 66.4 ± 8 years (range: 38 to 72 years), and 28% of the included patients were men. In all studies, HO was identified on postoperative radiographs. Among the nine studies that stratified the severity of HO,^8,11,13,14,18,20-22,24^ we identified five distinct classification systems,^11,13,14,20,24^ defined by Harwin, Dalury, Furia, Rader, and Toyoda. Heterogeneity of definitions and severity tiers among these HO classification systems precluded comparisons of HO severity across the study cohort. Without accounting for severity, the overall prevalence of HO ranged from 7.15% reported among studies using the Rader classification^20,21^ to 39.68% among studies using the Toyoda classification^24^ (Table 1).

**Table 1:** The Overall Rates of Heterotopic Ossification (HO) Among Studies That Used an Existing HO Classification System

Classification System	Studies Using Classification	HO	Knees	Rate of HO
Harwin	Harwin et al. and Barrack et al.	37	293	12.63%
Furia	Furia et al., Hasegawa et al., and Sterner et al.	62	510	12.16%
Rader	Rader et al. and Roth et al.	75	1049	7.15%
Toyoda	Toyoda et al.	25	63	39.68%
Dalury	Dalury et al.	76	500	15.20%

HO = heterotopic ossification

**Figure 2:**
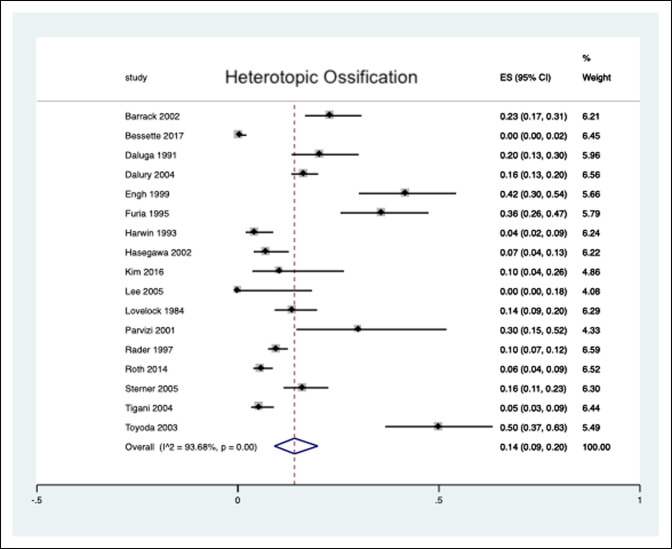
Forest plot depicting the overall prevalence of heterotopic ossification after primary total knee arthroplasty.

**Figure 3:**
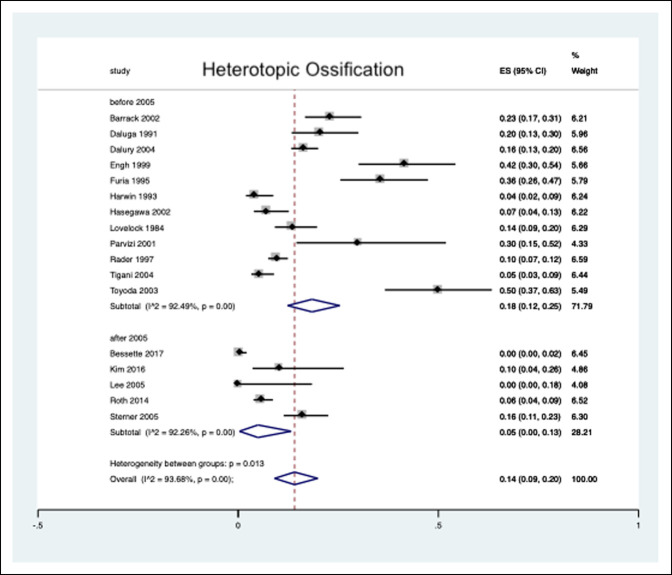
Forest plot depicting the prevalence of heterotopic ossification after primary total knee arthroplasty, stratified by articles published in the past 15 years compared with those published before then.

The pooled estimated HO prevalence in patients undergoing a primary TKA was 14% (9% to 20%) over an average follow-up of 40.1 months (11 to 108 months) (Figure 2). Owing to the wide range of publication years (1984 to 2017), we divided the literature into studies published before or after 2005 to determine whether a potential era effect exists for the reported presence of postoperative knee HO. Among the 17 included studies, five were published in the past 15 years^9,15,16,21,22^ and 12 before the year 2005.^8,10,11,12,13,14,17,18,19,20,23,24^ Our analysis revealed a pooled prevalence of 18% (12% to 25%) among studies published 15 or more years ago. In contrast, we identified a decreased pooled prevalence of 5% (0% to 13%) among studies published in the past 15 years (Figure 3).

## Discussion

This systematic review demonstrated that a relatively low rate of heterotopic ossification occurs after a primary TKA and that a need for a single, universally adopted classification system exists that characterizes the presence, extent, and location of HO. HO has been a strong topic of interest in the investigation of outcomes after TJA procedures. However, despite extensive reviews that have compiled HO prevalence after TJA for multiple other joints, this review is the first dedicated to TKA.

The exact pathogenesis of HO is not well understood but is generally believed to arise from mesenchymal stem cells driving an abnormally elevated degree of inflammation after tissue disruption.^1^ Many animal model and in vitro studies have been performed to investigate the cellular and molecular pathways that drive HO development, including cell types such as Tie2-expressing endothelial cells and release of molecular factors such as bone morphogenetic protein.^1^

Unsurprisingly, risk factors that have been reported for HO formation after TKA include hematoma formation, quadriceps tendon splitting, and retained bone particles after bone cuts, as well as patient-specific features such as genetics, a history of HO, and ankylosing spondylitis.^11,13,25^ From a clinical standpoint, most HO cases are discovered incidentally on follow-up radiographs. However, HO can occasionally become symptomatic and lead to reduced function and worse surgical outcomes. During the acute inflammatory stage of HO development, erythema, localized tenderness, and swelling may occur. Over time, the location and size of HO can potentially limit the range of motion (ROM) and joint function.^1^ This was recently demonstrated in a clinical study of revision TKA, in which a higher rate of preoperative HO was found in patients revised for stiffness compared with other aseptic causes. In addition, the most qualitatively severe HO cases were associated with a lower range of motion preoperatively and at every postoperative follow-up timepoint up to 1-year, indicating that severe HO formation is not fully mitigated even after revision TKA.^26^ Furthermore, when notable levels of functional debilitation are reached, revision surgery for HO excision may be required for symptom relief. For example, a recent systematic review of HO after total elbow arthroplasty found that 25% of patients who developed HO were symptomatic and, of those, 22% required subsequent revision surgery.^5^

We found that the overall prevalence of HO was 14% after primary TKA, which is comparable with the 10% rate reported after a total elbow arthroplasty.^5^ This rate is markedly lower compared with other TJAs because previous reviews of total ankle arthroplasty and cervical total disk arthroplasty found that 66%^4^ of patients and 44.6%^2^ of patients developed postoperative HO, respectively. However, the severity of HO, rather than just its presence, is most clinically relevant, with advanced/severe HO after cervical total disk arthroplasty reported to be 11.1%.^2^ Next, a review of total hip arthroplasty (THA) reported that 30% of patients developed HO postoperatively.^3^ Although this study only classified HO by the presence or absence of HO, the presence of HO was defined as any Brooker classification score greater than 0. For THA, the Brooker classification of HO severity is a well-established and widely used system for radiographic diagnosis of HO that is based on the degree of “bridging” between HO found in the soft tissues between the femur and pelvis.^27^

Stratification of HO after TKA based on severity was not possible because of the lack of a universal classification system for HO. Five different classification systems^11,13,14,20,24^ were used among the studies included in this review, which limits the comparability of studies evaluating HO after TKA. These classification systems differ in their consideration of size and location, as well as their correlation to clinical symptoms. The systems that specify location simply focus on the anterior femur and suprapatellar pouch region,^13,14,24^ except for Rader, which groups all nonanterior into the same grade.^20^ One system does not incorporate location at all and is strictly based on the total size of bone formation.^11^ Grading of size was also inconsistent, with only three actually quantifying size.^11,13,24^ Most importantly, only three systems had any correlation to clinical severity,^11,13,20^ such as decreased ROM or symptoms requiring surgery, whereas two had no association whatsoever.^14,24^ Notably, the Harwin^14^ classification was created from just 6 cases, none of which reported any pain or functional limitation secondary to HO. Thus, although the reported overall HO rates seem lower in TKA compared with other TJAs, definitive conclusions about the proportion of clinically notable HO arising in TKA cannot be made without a standardized, comprehensive HO severity classification system for TKA that includes clinical correlations. For example, if a high proportion of all HO cases observed are associated with notable discomfort or ROM restriction, early identification of HO radiographically may be essential to prevent these outcomes. In this scenario, greater consideration for more routine HO prophylaxis should be afforded to patients deemed to have a higher risk of HO, such as patients with a previous history of surgeries in the affected limb.

The two primary modalities of HO prophylaxis currently accepted are nonsteroidal anti-inflammatory drugs (NSAIDs) and radiation therapy.^1^ Both have been reported as effective measures for reducing postoperative HO prevalence, primarily in studies of THA, and this benefit has been shown to be superior when they are used in conjunction.^28^ NSAIDs are believed to be efficacious by inhibiting the differentiation of osteogenic precursor cells. In addition, they directly reduce prostaglandin formation, which is linked with differentiation of progenitor cells into osteoblasts that are ultimately the driver of ectopic bone formation.^1,28^ Radiation therapy is similarly designed on the concept of inhibiting the differentiation of osteogenic progenitor cells via DNA damage that curbs cell replication, with dosing typically falling between 400 and 800 cGy.^29^ Although NSAIDs are now commonly used for HO prophylaxis purposes, radiation is not as frequently used because of drawbacks of increased cost, risk of nonunion, wound healing complications, and other potential side effects, such as oligospermia and malignancy.^1^ However, preventive shielding measures have largely mitigated the risks of radiation, and long-term studies have supported the safety and efficacy of radiation therapy for HO prophylaxis.^28^ That being said, further studies with larger study populations are still required before definitively establishing it as a routine option.

Interestingly, there seems to be a time trend for HO prevalence, with decreased rates observed in more recent years. One plausible explanation for this is the increase in routine NSAID use over time.^1^ In North America, surgeon preference for venous thromboembolism (VTE) prophylaxis after arthroplasty has seen a notable shift toward aspirin over time.^30^ Postulated reasons for this increase in use include aspirin's cost effectiveness, favorable safety profile with low risk of postoperative hematoma formation and associated risk of persistent wound drainage or infection, additional role in pain management, and demonstrated effectiveness in VTE prevention.^31^ However, a simultaneous, potentially less recognized benefit of widespread postoperative use of aspirin is its role in the prevention of HO. As mentioned earlier, NSAIDs have been reported to be efficacious in the prevention of postoperative HO development. Thus, the increase in aspirin preference for VTE prophylaxis has also likely contributed to the lower rates of HO observed in studies published in the past 15 years.

Several limitations exist for this study. First, the nonrandomized observational nature of the included studies limits the generalizability of our results because of potential selection bias. In addition, the included studies contained notable methodologic heterogeneity, which could be attributed to different baseline and HO characteristics of the study populations included. In meta-analyses with high heterogeneity, funnel plot asymmetry is not as informative, and the Egger test cannot examine small study biases as conventionally suggested.^32^ Next, because of the limited amount of patient-level data provided by the studies included in this review, we could not perform additional analyses to identify the potential risk factors for HO. Finally, although we excluded studies that administered prophylactic radiation therapy, we could not account for NSAID use, especially given how commonly aspirin is used for VTE prophylaxis in contemporary lower extremity TJA, as discussed earlier. Thus, we aimed to address this limitation by analyzing the temporal effect on HO prevalence, finding a decreased HO rate in studies published more recently.

## Conclusion

Although the prevalence of HO after primary TKA seems to be low in comparison to rates reported for other TJA procedures, the assessment of TKA severity is unreliable in the absence of a single comprehensive, standardized classification system. HO does seem to have decreased in prevalence in more recent years, which is likely associated with the increased use of aspirin for routine VTE prophylaxis.
